# Trends in disease severity and quality of life outcome measures in pemphigus clinical trials: A scoping review

**DOI:** 10.1002/ski2.429

**Published:** 2024-07-24

**Authors:** Gaurav N. Pathak, Kush Patel, Christopher Wachuku, Thu Minh Truong, Priya Agarwal, Babar Rao

**Affiliations:** ^1^ Department of Dermatology Rutgers Robert Wood Johnson Medical School Piscataway New Jersey USA; ^2^ Department of Dermatology Rutgers New Jersey Medical School Newark New Jersey USA; ^3^ Department of Dermatology Rao Dermatology Atlantic Highlands New Jersey USA

## Abstract

Pemphigus represents a spectrum of autoimmune‐mediated blistering diseases associated with high morbidity, mortality and reduced quality of life (QoL). Despite an increase in pemphigus clinical trials, the varied instrument measurements of disease severity and QoL outcomes make comparisons between studies challenging. This study aimed to evaluate trends in the use of disease severity and QoL outcome measurements in pemphigus clinical trials. A review of pemphigus clinical trials was conducted using the PubMed, Embase, Cochrane Reviews and ClinicalTrials.gov databases up until September 2023. Only pemphigus randomized clinical trials that assessed at least one disease severity and/or QoL outcome were included. Overall, 53 clinical trials were eligible for this review. All clinical trials evaluated a disease severity outcome, with the Pemphigus Disease Area Index being the most used validated questionnaire (28.3% of trials) and more popular after 2015 (47.8% of trials since). The autoimmune bullous skin disorder intensity score (7.6%) and visual analogue measurements (7.6%) have fallen out of favour. Most studies now include lab parameters (56.5% of trials after 2015), with anti‐desmoglein 1 and 3 antibody levels (30.2%), immunoglobulins (IgG and/or IgM and IgA) (11.3%), and anti‐drug antibody levels (7.6%) being frequently evaluated. A small portion of trials evaluated QoL (26.5% of studies), with the autoimmune bullous quality of life being the most common (15.1%), however QoL utilization as an outcome measure has been increasing since 2015 (61.1% of trials since). Standardising the use of validated outcome measurements allows for better data interpretation, comparability and clinical application of results.

**What is already known?**

- Pemphigus is a group of potentially life‐threatening autoimmune‐mediated blistering diseases with high mortality and reduced quality of life (QoL).
- Common clinical efficacy outcome measures include disease control, relapse prevention and reduced dependency on corticosteroids.

**What does this study add?**

- The use of standardized outcome measurements including the pemphigus disease area index and anti‐desmoglein 1 and 3 levels have been increasing longitudinally, while the autoimmune bullous skin disorder intensity score has declined in favourability.
- Despite the profound impact on QoL, only 26.5% of trials included a QoL measurement as an outcome measurement, with the autoimmune bullous quality of life being most commonly used.

## 1 INTRODUCTION

Pemphigus is a group of rare, chronic autoimmune blistering diseases characterised by mucocutaneous lesions and easily rupturing cutaneous bullae affecting nearly 5.2 per 100 000 people in the United States.^1^ Autoantibody production against desmogleins, a family of desmosomal cadherins involved in cell‐to‐cell adhesion of keratinocytes, results in blister formation. The two most common subtypes of pemphigus include pemphigus vulgaris (PV), which primarily targets desmoglein 3 with mucosal involvement, as well as pemphigus foliaceous (PF), which primarily targets desmoglein 1, generally without mucosal involvement.^2^, ^3^ Pemphigus is associated with high morbidity and can be fatal due to severe blistering, secondary infections and malnutrition, with mortality rates up to 5–15% yearly.^4^ Current treatment options for pemphigus include systemic glucocorticoid therapy, immunosuppressive drugs and targeted immunotherapy with anti‐B cell monoclonal antibodies such as rituximab.^3^ Despite new therapeutic advances, long‐term pemphigus treatment remains an unmet medical need. Pemphigus patients often suffer increased risks of infection due to prolonged steroid use, and disease severity fluctuates due to disease progression/relapse, thereby necessitating the need for objective disease scoring systems.

With limited long‐term efficacious treatment options, the variance in outcome measures in pemphigus clinical trials makes therapeutic comparisons between studies challenging. Additionally, pemphigus is associated with high physical, emotional and social distress with as high as 77% of patients experiencing anxiety and depression.^5^, ^6^ Quality of life (QoL) assessments in pemphigus clinical trials may aid in characterising treatment efficacy and aid in the representation of patient‐reported goal‐concordant care. As new‐targeted therapies are emerging, pemphigus clinical trials are increasing.^7^ As these studies are being increasingly conducted, there is a greater need for standardisation of validated measurement tools to assess clinical endpoints.

Two such tools include the autoimmune bullous skin disorder intensity score (ABSIS) and the Pemphigus Disease Area Index (PDAI), which are comprehensive scoring tools used to evaluate pemphigus disease severity.^8^ Furthermore, serum testing is increasingly utilised to assess pemphigus disease severity. Particularly, anti‐desmoglein‐1 and anti‐desmoglein‐3 antibody levels can be used to distinguish pemphigus subtypes, characterise disease progression, and indicate response to treatment, though they should not be utilised in isolation. Immunoglobulins (particularly IgG levels and/or IgM and IgA) can also be used to assess pemphigus severity, and anti‐drug antibodies allow for the monitoring of treatment efficacy and development of drug resistance.^9^, ^10^

Other measures, such as investigator global assessment (IGA), disease activity index, the dose of corticosteroid and time until remission are also used. The utilization of dermatology‐specific questionnaires, such as the Dermatology Life Quality Index (DLQI) and autoimmune bullous quality of life (ABQOL) may aid in capturing pemphigus‐specific psychosocial effects.

The use of objective and subjective outcomes via a diverse subset of scales and questionnaires makes the compilation of data and intra‐study cross‐comparisons challenging, particularly in rare diseases such as pemphigus where the number of patients is limited. Utilization of instruments that are not validated may hinder study legitimacy and inadequately capture pemphigus clinical outcomes. Given the role of clinical research as evidence for informed clinical decision‐making, analysing trends in pemphigus disease severity and QoL measures is necessary. Assessing the current landscape of these measuring tools may guide future questionnaire/tool selection in future clinical trials to allow for improved compilation of data for more informed therapeutic decision‐making for clinicians. In this scoping review, we aim to analyse trends in disease severity and QoL tools and measures in past and current pemphigus clinical trials.

## 2 METHODS

A comprehensive search of past and current pemphigus clinical trials was conducted using PubMed, Embase, Cochrane Reviews and Clinicaltrials.gov databases up to 1 September 2023. Inclusion criteria included: randomized clinical trials evaluating pemphigus (any subtype), published in English, and studies must have reported at least one disease severity or QoL outcome. Exclusion criteria: studies that are not in English, not for pemphigus, not assessing at least one QoL/disease severity measure and familial pemphigus studies. Additionally, phase 1, pharmacokinetics, animal studies and “proof of concept” clinical studies were excluded since capturing disease severity, and QoL measures may not be reasonable or expected in these study types. Additionally, only studies with complete outcome measurements data were included.

### 2.1 PubMed search

A medical literature search of the PubMed database was conducted using the search terms: ((((“pemphigus”[MeSH Terms]) OR (“pemphigus”[Title/Abstract])) OR (“PV”[Title/Abstract])) OR (“PF”[Title/Abstract])) AND ((((Severity of Illness Index[Title/Abstract]) OR (“QoL”[Title/Abstract])) OR (autoimmune bullous skin disorder intensity score[Title/Abstract])) OR (“QoL”[MeSH Terms])). Studies were populated from 1985 (earliest relevant study) to 2023.

### 2.2 Embase search

A comprehensive search for pemphigus clinical trials was conducted using the following search term in Embase: ((((“pemphigus”[MeSH Terms]) OR (“pemphigus”[Title/Abstract])) OR (“PV”[Title/Abstract])) OR ("PF”[Title/Abstract])) AND ((((Severity of Illness Index[Title/Abstract]) OR (“QoL”[Title/Abstract])) OR (autoimmune bullous skin disorder intensity score[Title/Abstract])) OR (“QoL”[MeSH Terms])).

### 2.3 Cochrane libraries

A search of Cochrane reviews was conducted using “pemphigus” with no other filters or Boolean operators.

### 2.4 Clinicaltrials.gov

Clinical trials of any “status” of recruitment were included, and clinical trials of any age and sex group were also eligible. Studies with and without results were included, and only phase 2–4 clinical trials were evaluated. The search term “pemphigus” was applied with “interventional studies, phase 2, phase 3, phase 4, and phase not applicable” filters were applied.

After the abstraction and selection of studies, duplicates were removed, and full‐text review was conducted to ensure the selected studies were applicable to the study inclusion criteria. The parameters of interest included specific indication, clinical trial current status, year trial posted, enrolment (or projection), patient age group in trial (adult or paediatric), gender of included patients, phase of trial, intervention type (pharmaceutical or procedural), specific intervention, geographic location of trial (US or outside US), disease severity collected (yes/no), number of outcomes collected, what specific disease outcome tool was utilised, lab collected as outcome measure (yes/no), type of lab collected, QoL collected (yes/no), number of QoL instruments used (if applicable) and what specific QoL tool was utilised. Data were extracted and consolidated onto a separate Excel sheet for the variables included and all parameters collected were cross‐checked by two authors (G.N.P. and K.P.) independently, and any discrepancies were resolved by consensus. Data were aggregated and summarised in tables and longitudinal endpoints were described using visuals.

## 3 RESULTS

After study randomisation, a total of 169 studies from PubMed, 40 from clinicaltrials.gov, 12 from Cochrane libraries and 259 results from Embase were retrieved. After the removal of studies that (1) were not randomized clinical trials, (2) were pharmacokinetic/animal/proof of concept studies, (3) were not written in English, (4) did not discuss pemphigus and (5) were duplicates, there were a total of 53 eligible studies for this review (Table 1).

**TABLE 1:** Summary of search strategy for eligible studies.

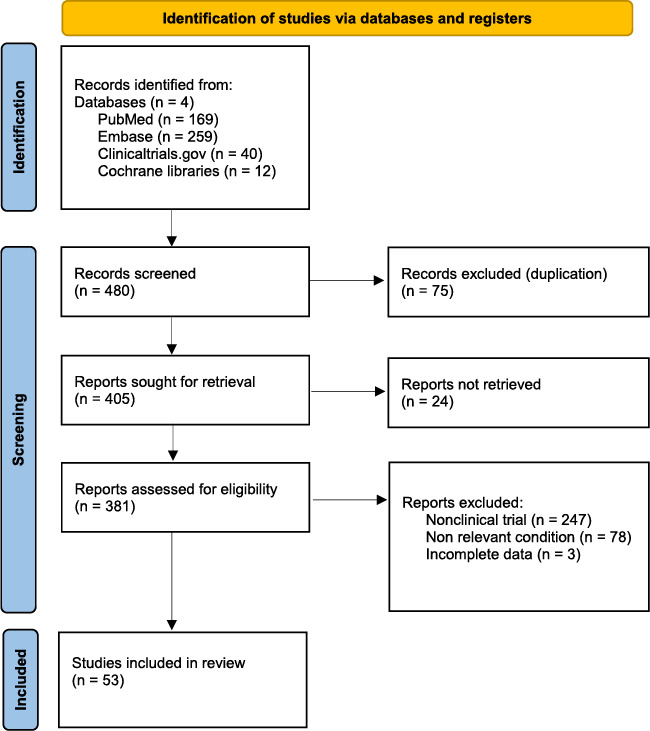

A total of 53 eligible pemphigus clinical trials were evaluated, of which 49.1% were for PV, 41.5% PV/foliaceus and 7.5% for mucosal pemphigus (Table 2). Most studies (51/53) were for adults and there was an equal distribution of included phase 2 and phase 3 trials, although most trials were unspecified (41.5%). All 53 of the included studies recorded some aspect of a disease severity outcome measure; however, only 26.5% of the studies evaluated a QoL outcome. Later phase trials (phase 3 and 4) had higher rates of collecting pemphigus QoL outcomes, and the number of pemphigus clinical trials have been increasing since 2006. A vast majority of the studies (50/53) evaluated a pharmaceutical intervention for pemphigus, while the remaining three measured a procedural or device intervention. The trial sizes ranged anywhere from 1 patient to 222 patients, with the later phase trials generally consisting of larger patient cohorts. Approximately 19 of the selected trials were conducted in the United States, and 26 were performed worldwide (8 were unknown). Lab parameters were more commonly reported as an outcome measure than QoL measures, with 39.6% (21/53 trials) of pemphigus clinical trials assessing a lab parameter as a primary or secondary outcome. Disease severity measures were always collected as at least one primary outcome measure, and QoL measures/lab parameters were usually secondary measures.

**TABLE 2:** Characteristics of included pemphigus clinical trials.

Number of clinical trials by indication (*n* = 53)	Number of clinical trials by age groups (*n* = 53)	Breakdown by study type (*n* = 53)	Percent of trials assessing disease severity by trial type (*n*, %)	Percent of trials that assessed a QoL by trial type (*n* = 14)	Number of trials per year range
PV = 26 Pemphigus (both) = 22 MP = 4 PF = 1	Adult only: 51 Children + adult: 2	Phase 2: 13 (24.5%) Phase 3: 13 (24.5%) Phase 4: 2 (3.8%) Multiple: 3 (5.7%) Unspecified: 22 (41.5%)	Phase 2: 13 (100%) Phase 3: 13 (100%) Phase 4: 2 (100%) Multiple: 3 (100%) Unspecified: 22 (100%)	Phase 2: 3 (23.1%) Phase 3: 5 (38.5%) Phase 4: 2 (100%) Multiple: 0 Unspecified: 4 (18.2%)	1985–1995: 5 1996–2000: 2 2001–2005: 5 2006–2010: 12 2011–2015: 8 2016–2020: 17 2021–2023: 4

Abbreviations: MP, mucosal pemphigus; PF, pemphigus foliaceus; PV, pemphigus vulgaris; QoL, quality of life.

A total of 55 different disease severity outcome measures were identified in this review, and the most commonly measured outcomes were adverse events (AEs) (49.1%), rate of complete response/remission (39.6%) and cumulative steroid dose (37.7%) (Table 3). The most commonly utilised pemphigus disease severity validated measurement were the PDAI (15/53, 28.3%) followed by the ABSIS (4/53, 7.6%), and visual analogue measurement scales (4/53, 7.6%). PDAI became especially popular after 2015, in which 11/23 (47.8%) of the studies utilised it. ABSIS and visual analogue scale measurement have remained relatively low in use since their inception in 2007, with no increases in use longitudinally. Despite the lack of standardized pemphigus grading systems, older studies (before 2000) evaluated time until clinical response (4/7), which was largely replaced with the rate of complete response/remission (28/31 of studies after 2010). However, some endpoints, such as the rate of cumulative steroid dose (4/12 before 2005), still remain in use today (10/31 since 2010).

**TABLE 3:** The frequency and proportion of selected disease severity outcome measures.

Disease severity outcome measurement	Frequency of use in studies	Percent use in studies, *n* = 53
Adverse effects	26	49.06%
Complete remission/response rate	21	39.62%
Cumulative steroid dose	20	37.74%
Time to disease control	18	33.96%
PDAI	15	28.30%
Treatment response rate (partial/complete)	12	22.64%
Relapse/flare	12	22.64%
Time to relapse	12	22.64%
Serious AEs	8	15.09%
Duration of remission	6	11.32%
Weeks in remission	4	7.55%
Reduction in steroid dose	4	7.55%
ABSIS	4	7.55%
VAS	4	7.55%
CDA	3	5.66%
Sustained remission	3	5.66%
Prevent new lesions	3	5.66%
Area of skin healed	3	5.66%
Duration of steroid use	3	5.66%
Number of recurrences	3	5.66%
Time to remission	3	5.66%
Number of infections	2	3.77%
Pain severity	2	3.77%

Abbreviations: ABSIS, autoimmune bullous skin disorder intensity score; AE, adverse events; CDA, Control of disease activity; PDAI, pemphigus disease area index; VAS, Visual analogue scale measurement.

**FIGURE 1:**
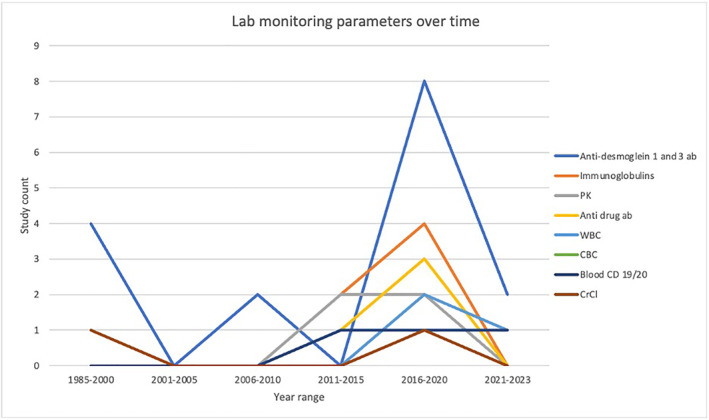
Lab‐monitoring parameters over time.

**FIGURE 2:**
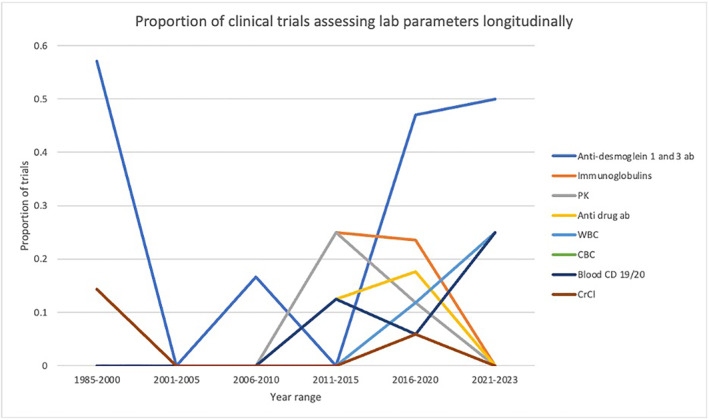
Proportion of clinical trials assessing lab parameters longitudinally.

Lab monitoring was often collected in pemphigus clinical trials, with anti‐desmoglein 1 and 3 levels being assessed in 28.3% of clinical trials, and immunoglobulins (IgG and/or IgM and IgA) (11.32%) and drug pharmacokinetics (9.4%) being frequently assessed as well (Table 4). In general, the collection of lab parameters as primary or secondary endpoints has increased since 2015, with 13/23 (56.5%) of the studies conducted during that time period evaluating a lab monitoring parameter (Figure 1). Anti desmoglein 1 and 3 antibodies levels have been the most commonly collected parameters longitudinally; however, the use of immunoglobulins, anti‐drug antibody levels and blood CD19/20 counts has been increasing since 2015 (Figure 2).

**TABLE 4:** The frequency and proportion of laboratory‐monitoring parameters.

Lab parameter	Frequency of use in studies	Percent of use in studies, *n* = 53
Anti‐desmoglein 1 and 3 ab	16	30.19%
Immunoglobulins (IgG and/or IgM and IgA)	6	11.32%
PK conc	4	7.55%
Anti‐drug antibody	4	7.55%
WBC	3	5.66%
CBC	2	3.77%
Blood CD 19/20	3	5.66%
CrCl	2	3.77%

Abbreviations: CBC, complete blood counts; CrCl, creatinine clearance; PK, pharmacokinetics; WBC, white blood cell counts.

**FIGURE 3:**
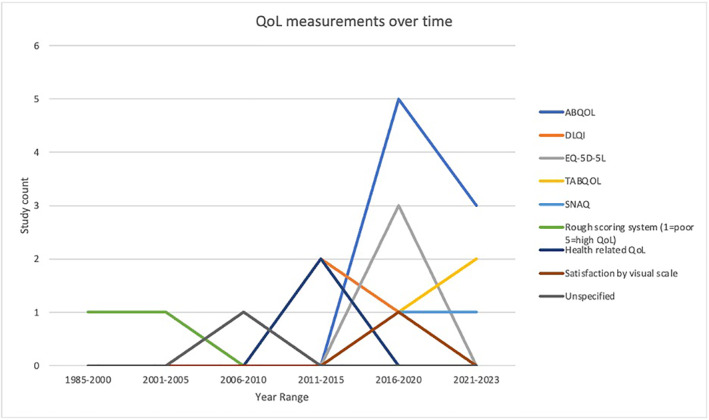
QoL measurements over time. QoL, quality of life.

**FIGURE 4:**
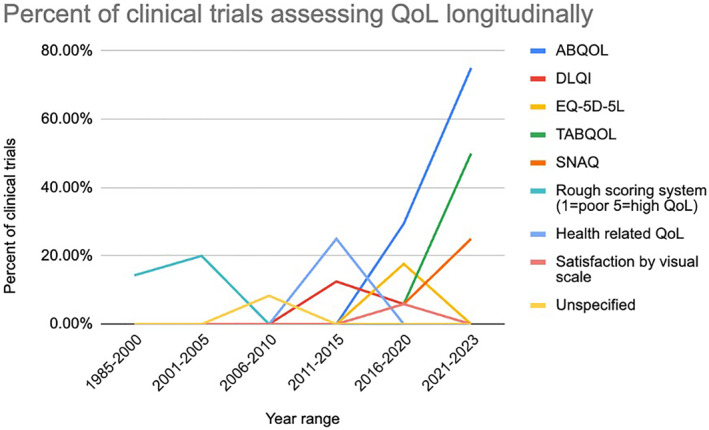
Percent of clinical trials assessing QoL longitudinally. QoL, quality of life.

Information regarding QoL was also collected, and 8 different QoL measurement tools were used in the selected pemphigus clinical trials. Of the 53 trials, 14 (26.5%) included a QoL measurement in their design, with the ABQOL (57.14% of studies using QoL) being the most common, followed by the DLQI (28.6%) and EQ‐5D‐5L (21.43%) (Table 5). The use of validated QoL measurement tools increased rapidly after 2011–2015, whereas before that time the use of QoL measurements in clinical trials was low, with primarily invalidated scoring systems (rough scoring systems) (Figures 3 and 4). Since 2011, the use of ABQOL and treatment autoimmune bullous disease quality of life (TABQOL) have been increasing rapidly, accounting for 11/18 (61.1%) of the tools used in clinical trials from 2016 onwards.

**TABLE 5:** Frequency and proportion of quality of life outcome measures.

QoL measure	Frequency of use in studies	Percent of use, *n* = 53
ABQOL	8	15.09%
EQ‐5D‐5L	3	5.66%
TABQOL	3	5.66%
DLQI	2	3.77%
SNAQ	2	3.77%
Rough scoring system (1 = poor 5 = high QoL)	2	3.77%
Health‐related QoL	2	3.77%
Satisfaction by visual scale	1	1.89%
Unspecified	1	1.89%

Abbreviations: ABQOL, autoimmune bullous disease quality of life; DLQI, dermatology quality life index; Eq‐5D‐5L, EuroQol 5 Dimension 5 Level; SNAQ, simplified nutritional appetite questionnaire; TABQOL, treatment autoimmune bullous disease quality of life.

## 4 DISCUSSION

Pemphigus continues to represent a disease management challenge, and clinical trials aimed at evaluating key treatment outcomes do not yet have a universal set of measures. There was a large degree of variability of the disease severity clinical endpoints highlighting the heterogeneity of pemphigus clinical trials and lack of outcomes standardisation. However, a clear trend was seen in PDAI as a frequently used measure of severity since 2015. Furthermore, time until remission/response has been increasing in recent years highlighting a common endpoint in pemphigus trials. For lab data collected, anti‐desmoglein 1 and 3 antibodies are the most common measures with immunoglobulins (IgG and/or IgM and IgA) and blood CD19/20 usage on the rise. In addition to severity and lab measures associated with pemphigus, some QoL data were also collected using trials, but many studies did not screen for QoL. Of the studies that did include QoL statistics, ABQOL was the highest in usage with a frequency of 8 studies (Table 5).

### 4.1 Disease severity

There are three major clinical pemphigus disease activity indexes: PDAI, ABSIS, and PVAS; however, their routine use in clinical trials did not begin until recently. The PDAI is a questionnaire that assesses the number of erosions, blisters and erythema in 12 anatomic locations with scores 0 (absent) to 10 (>3 lesions or at least one lesion >16 cm) for skin, scalp (counts as 1 anatomic location), and mucous membranes (max score 250). Damage is also calculated as 0 (absent) or 1 (present) for the skin and scalp for a maximum score of 13.^8^ Advantages of the PDAI include that it has the highest validity among the three validated disease severity indexes.^11^ Its scores represent true clinical severity, it can also be used for treatment monitoring, and it has more reproducible scoring than ABSIS for further clinical effectiveness.

Due to its extensive grading of various anatomic locations, PDAI may be able to better detect smaller changes in disease severity in mild to severe cases and assess treatment responders more effectively. Disadvantages include the length of the questionnaire: despite being 32 total questions versus 43 for ABSIS, the mean length of time to complete the questionnaire was 4.7 versus 3.9 min (ABSIS).^8^, ^12^ The ABSIS scoring system was developed in 2007 which uses the rules of 9's to estimate the percentage of skin blistering (BSA), oral involvement, and quality of blisters (factor of discomfort and severity). ABSIS may be advantageous in that it provides both qualitative and quantitative information; however, variances in internal validity and low use in clinical trials (only 4/53) suggest that it may have fallen out of favour.^13^ Other disease severity scoring methods include the pemphigus area and activity score, which divides the body into four areas and considers new blisters, current blisters and percent area. PAAS was not utilised in any trial that was evaluated, possibly due to its limitation in BSA utilization and clinical relevance of counting the correlation of blisters with symptoms/mortality.^14^ Other general measures, such as visual analogue scale, IGA and patient global assessment are not routinely used. The increase in PDAI longitudinally may allow for better data compilation and cross‐study comparisons.

Adverse effect reporting, objective response rate and time to disease control were all more commonly reported than validated measures. However, these endpoints are generally non‐specific measures of all phase 2–3 clinical trials that assess clinical efficacy and safety endpoints and are generally expected to largely stay the same over time.^15^ Cumulative steroid use is another outcome measure; however, various factors including medication adherence, physician prescribing habits, comorbidities, labs and contraindications may complicate its accuracy as an objective metric.^16^

### 4.2 Lab monitoring

Assessing lab parameters as a clinical endpoint has become an increasingly important metric used in pemphigus clinical trial and in clinical therapeutic decision‐making. The underlying pathogenesis of pemphigus is characterised by the loss of epidermal cell adhesion caused by autoantibody (IgG) production to desmosomal adhesion proteins, particularly desmoglein 1 (Dsg1) and desmoglein 3 (Dsg3).^17^ Thus, immunoglobulins (IgG and/or IgM and IgA) and anti‐Dsg titres have been a mainstay of evaluating disease course and treatment efficacy, and that is reflected in their common and increasing use in clinical trials, particularly since 2015. Other measurements, such as anti‐drug antibodies have also been collected recently and may correlate with treatment resistance.^18^ Regular evaluation of these monitoring parameters and other labs (CBC/CMP) may aid in treatment selection, guide treatment monitoring and evaluate disease progression objectively.

### 4.3 Quality of life

For many patients, the appearance of large flaccid fluid filled blisters and subsequent hypopigmentation post treatment can alter the skin appearance and affect self‐esteem. Additionally, long‐term corticosteroid use can further affect patient appearance, causing significant psychological trauma to patients.^19^ Pemphigus patients have a 3–4 times higher prevalence of psychiatric comorbidities.^19^ Despite significant physical, emotional, and social distress, QoL measurements were not routinely captured in pemphigus clinical trials (26.5%). The most commonly used measures include the ABQOL/TABQOL, EQ‐5D‐5L and DLQI. These are all validated QoL measures; EQ‐5D‐5L and DLQI are general dermatology questionnaires and ABQOL/TABQOL are specific to autoimmune blistering diseases.^20^

The DLQI and EQ‐5D‐5L are dermatology‐specific QoL tools that evaluate psychosocial impacts of dermatologic conditions and have been extensively studied in other conditions including psoriasis, alopecia and vitiligo.^21^ Specifically, the DLQI is a 10‐question survey that assesses physical, psychological and social aspects of QoL through a series of dermatology‐focused disease effects. High DLQI scores equate to high QoL impairment (range 0–30).^22^ Advantages to using these general dermatology QoL are that they have great established validity, have greater awareness and widespread use and they allow for easier cross‐comparisons between pemphigus and other dermatologic diseases.^23^ Additionally, they are short questionnaires that are simple and quick to complete, with a high success rate of accurate completion. However, utilization of general dermatology questionnaires such as the DLQI may not adequately address the disease‐specific concerns of pemphigus patients.

Development of ABQOL in 2012 introduced a 17‐item, self‐reported questionnaire to assess QoL and was found to have moderate correlation with DLQI in validity but was more sensitive than DLQI in discriminant validity.^24^ Likewise, the TABQOL questionnaire is an AIBD‐specific metric that is a patient‐centred tool to evaluate the treatment‐specific impact of QoL in specific groups (it also has high correlation with ABQOL and DLQI).^25^ Both of these AIBD‐specific metrics may better capture pemphigus patients' unique concerns and may gauge treatment progress. Since its inception in 2012, ABQOL has been increasing in use and the overall utilization of QoL in clinical trials has increased. In clinical trials before 2010, utilization of general QoL was more limited and conducted using general patient‐reported subjective measures. This may be due to the recent recognition, awareness, and development of QoL as an important clinical instrument for patient evaluation and treatment.^26^ We find similar trends of more specific QoL measurements being used similar to studies in hidradenitis suppurativa and other dermatologic conditions.^26^, ^27^

### 4.4 Clinical focus/limitations

In rare diseases with limited treatment options, selection of the optimal agent backed by the most robust level of evidence is crucial in improving clinical outcomes. Utilization of validated disease severity and QoL instruments in clinical trials can allow clinicians to better compare and compile data regarding optimal agent selection.^27^ In pemphigus, utilization of disease‐specific evaluation tools such as the PDAI, ABSIS and ABQOL in concert with lab monitoring for anti‐Dsg 1 and 3 antibodies may best reflect disease‐specific concerns. Utilization of these measures may allow clinicians to better monitor treatment efficacy, evaluate disease progression and provide patient goal‐concordant care through regular incorporation in their disease management.^28^ Increasing awareness of these tools and working towards a standardized approach in clinical practice and clinical trials may aid in better data interpretation, comparability and clinical application of results worldwide.

Limitations to this review are that it only included pemphigus clinical trials and excluded other publication types including cohort studies, case–control studies, cases series, and case reports. However, clinical trials often represent the highest level of medical evidence and are a robust study type in guiding therapeutic decision‐making, and multiple databases were utilised to ensure a broadened scope of clinical trial inclusion.^29^ Inadequate representation of pemphigus subtypes, including pemphigus vegetans, IgA pemphigus and paraneoplastic pemphigus may limit the generalisability of these findings; however, PV and PF were the most common indications in evaluated clinical trials and are the most common variants worldwide. The low frequency of QoL use in pemphigus trials makes evaluating trends in QoL more challenging, although the overwhelming majority of studies used ABQOL. We also find inconsistencies in the definition of some outcome measures, such as flare/recurrence. There should be standardisation of this definition that allows for greater consistency across trials and more accurate data comparison and interpretation. Ultimately, future studies should evaluate multiple validated disease severity and QoL measures to further establish scale utility, validity and applicability.

## 5 CONCLUSION

Pemphigus represents a spectrum of autoimmune blistering diseases associated with high mortality/morbidity and significant reductions in QoL. Utilization of PDAI has increased significantly since 2015 and is the most widely used validated disease severity outcome metric, and disease‐specific lab monitoring parameters such as anti‐desmoglein 1,3 antibodies and immunoglobulin levels (primarily IgG, and/or IgM and IgA) have also increased. Non‐specific measures such as AEs, objective response rate and corticosteroid cumulative use remain a mainstay of pemphigus assessment. These measures may help objectively monitor treatment efficacy and disease progression clinically. Ultimately, utilization of questionnaires assessing disease severity, such as PDAI, in concert with lab monitoring of anti‐desmoglein 1 and 3 antibodies may best allow for a holistic evaluation of disease severity, progression, and outcomes in pemphigus clinical trials.

Pemphigus‐specific QoL measurements such as ABQOL may better capture disease‐specific concerns as opposed to general dermatology QoL (DLQI) measures. Limiting the number of disease severity and QoL questionnaires in use and standardising implementation can aid in assessing outcome measures across clinical trials and allow for better data interpretation, comparability and clinical application of results. Given the potential for relapse in these patients, it is recommended that disease severity, QoL measurements, lab monitoring and utilization of questionnaires can be repeated at regular intervals to monitor patients on a long‐term basis.

## CONFLICT OF INTEREST STATEMENT

Dr. Rao is a speaker for Incyte and Amgen. The authors declare no conflicts of interest.

## AUTHOR CONTRIBUTIONS

**Gaurav N. Pathak**: Conceptualization (equal); data curation (equal); formal analysis (equal); investigation (equal); methodology (equal); validation (equal); writing – original draft (equal); writing – review & editing (equal). **Kush Patel**: Conceptualization (equal); data curation (equal); formal analysis (equal); investigation (equal); methodology (equal); resources (equal); writing – original draft (equal); writing – review & editing (equal). **Christopher Wachuku**: Conceptualization (supporting); data curation (supporting); formal analysis (supporting); investigation (supporting); methodology (supporting); validation (equal); writing – original draft (equal); writing – review & editing (equal). **Thu Minh Truong**: Conceptualization (supporting); data curation (supporting); investigation (supporting); methodology (supporting); resources (equal); validation (equal); writing – original draft (equal); writing – review & editing (equal). **Priya Agarwal**: Writing – review & editing (equal). **Babar Rao**: Conceptualization (equal); investigation (equal); methodology (equal); supervision (lead); writing – original draft (equal); writing – review & editing (equal).

## ETHICS STATEMENT

Not applicable.

## PATIENT CONSENT

Not applicable.

## Data Availability

All data utilized in this study are publicly available.
